# High mobility group Box-1 inhibits cancer cell motility and metastasis by suppressing activation of transcription factor CREB and nWASP expression

**DOI:** 10.18632/oncotarget.2150

**Published:** 2014-06-30

**Authors:** Zhenghong Zuo, Xun Che, Yulei Wang, Bowen Li, Jingxia Li, Wei Dai, Charles P. Lin, Chuanshu Huang

**Affiliations:** ^1^ Nelson Institute of Environmental Medicine, New York University School of Medicine, Tuxedo, NY; ^2^ State Key Laboratory of Cellular Stress Biology, School of Life Sciences, Xiamen University, Xiamen, China; ^3^ Mouse Cancer Genetics Program, Center for Cancer Research, National Cancer Institute-Frederick, Frederick, MD, USA

**Keywords:** HMGB1, metastasis, CREB, nWASP

## Abstract

The ability to metastasize is a hallmark of malignant tumors, and metastasis is the principal cause of death of cancer patients. The High Mobility Group Box-1 (HMGB1) is a multifunction protein that serves as both a chromatin protein and an extracellular signaling molecule. Our current study demonstrated a novel mechanism of HMGB1 in the regulation of cancer cell actin polymerization, cell skeleton formation, cancer cell motility and metastasis. We found that knockdown of HMGB1 in human lung cancer A549 cells significantly increased cell β-actin polymerization, cell skeleton formation, cancer cell migration and invasion *in vitro*, as well as metastasis *in vivo*. And this increase could be inhibited by treatment of HMGB1 knockdown cells with recombinant human HMGB1. Further studies discovered that HMGB1 suppressed phosphorylation, nuclear translocation, and activation of CREB, by inhibiting nuclear translocation of PKA catalytic subunit. This reduces nWASP mRNA transcription and expression, further impairing cancer cell motility. Our findings on the novel mechanism underlying the HMGB1 anti-metastatic effect on cancer provides significant insight into the understanding of the nature of HMGB1 in cancer invasion and metastasis, further serving as key information for utilization of HMGB1 and its regulated downstream components as new targets for cancer therapy.

## INTRODUCTION

Cancer metastasis, one of the major reasons for failure of cancer patient therapy, involves complex processes, including basement membrane degradation, cell migration, stromal invasion, angiogenesis, intravasation, adhesion, extravasation, and colonization [1, 2]. These processes are regulated by numerous metastasis promoting and suppressing genes [3]. Thus, identifying novel metastasis-regulated genes, particularly those acting on cell movement, as well as elucidating the mechanisms of their interactions, may provide new insights into the pathogenesis and management of cancer. One of the most important cell structures involved in cell metastasis is the microfilament, which functions in cytokinesis, amoeboid movement, and cell body shape change, in quite a versatile manner [4]. Actin is the globular protein that forms microfilaments, which play an important role in cell migration. The reorganization of actin proteins is the main force driving the cell movement [5]. Various studies have proven that actin polymerization mediates the cancer progression by altering cell motility [6]. Actin reorganization is well regulated by different proteins, including Wiskott–Aldrich syndrome protein (WASP) and its homologous form expressed in neurons, nWASP, which are both actin binding proteins. The second WASP-homology (WH2) domain and cofilin-homology sequence (CHS) domain, located at C-terminal region of nWASP, is responsible for actin binding and nWASP's function in regulation of cell motility [7]. It has been discovered for long that actin would serve as potential cancer drug target due to its importance in cell mobility [8]. And in recent study, nWASP has been revealed as one of the signaling molecules linking actin dynamics to delamination, a cellular process with potential association with cancer cell migration

HMGB1, also known as amphoterin, is a highly conserved nuclear protein which acts as a nuclear factor and enhances interaction between DNA and its binding proteins by bending the DNA[9]. In this case, one of HMGB1's important roles is to recruit the binding of p53/p73 transcription complexes to DNA, promoting transcription of the p53/p73-dependent gene [10]. The extranuclear role of HMGB1 mainly acts as a cytokine, and functions extracellularly after it has been passively released from necrotic cells or actively secreted by inflammatory cells, for example, in activated macrophages [11, 12]. HMGB1 has high affinity with several proteins to form different complexes and interacts with various receptors such as Receptor for Advanced Glycation Endproducts (RAGE), and Toll-like receptors (TLR)-2, TLR-4, TLR-9, and CD24 [13-15]. These interactions participate in the cellular response to infection and injury, and are associated with inflammation. But their roles in cancer metastasis is not clear. This led us to expand our investigation of HMGB1's regulatory abilities on cancer cell motility and metastasis.

## RESULTS

### HMGB1 Inhibited Cell Migration, Invasion and Metastasis *in vitro* and *in vivo*

**Figure 1:**
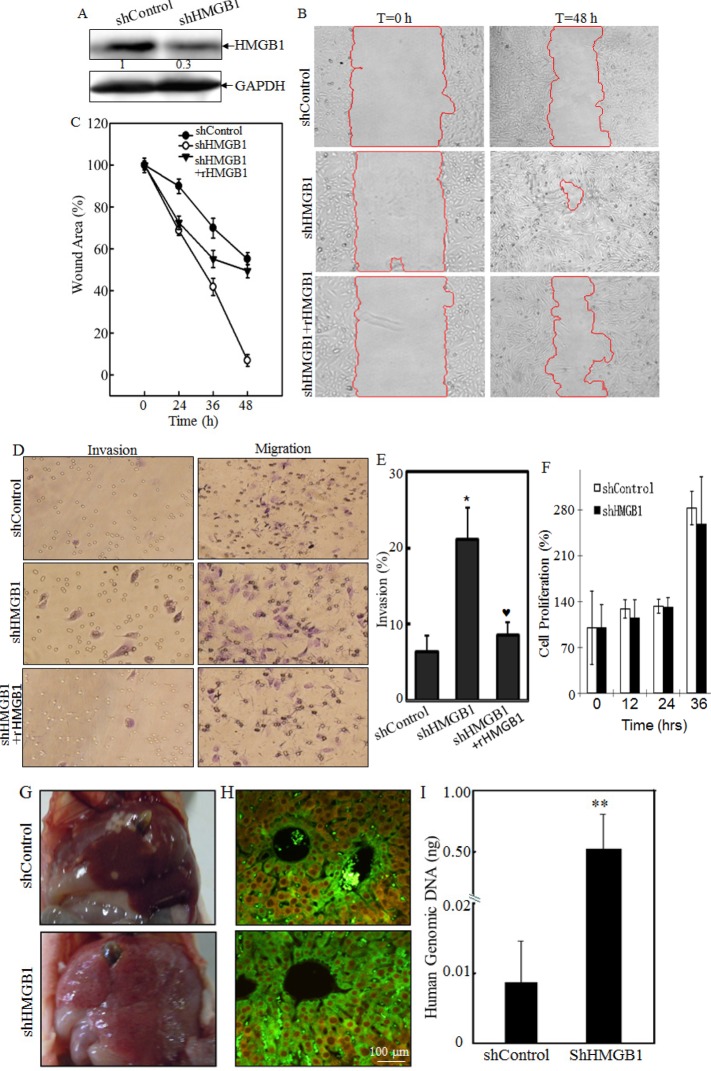
Knockdown of HMGB1 increased lung cancer A549 cell migration, invasion *in vitro* and liver metastasis *in vivo* A) Knockdown of HMGB1 expression in A549 cells with stably transfection of HMGB1 shRNA was identified by Western Blotting. B) Cell migration behavior of A549(shControl) and A549(shHMGB1), A549(shHMGB1+rHMGB1) were evaluated by wound-healing assay. Photographs were taken at specific time points as indicated. C) The wound area was quantified using cell migration analysis software and the results were presented as indicated. D) Invasion abilities of A549 (shControl), A549 (shHMGB1) and A549 (shHMGB1+rHMGB1) were determined using BD BioCoat^TM^ Matrigel^TM^ Invasion Chamber. The migration abilities was determined using the same system except that the matrigel was not applied. E) Results were expressed as the mean percentage ± S.D. of invasive cells from at least three independent experiments. F) A549 (shControl) and A549 (shHMGB1) were cultured in each well in a six-well plates for 72 hours. Cell proliferation was measured and results are expressed as percentage of time 0; Values are means ± SE in triplicate. G) A549 (shControl) and A549 (shHMGB1) cells injected into the mouse tail veins of 4-week-old female nude mice. After 4 weeks of the injection, the mice were sacrificed under euthanized with diethyl ether, and the liver tissues were removed and examined microscopically for metastatic foci. H) After fixation in 10% formalin solution and tissue from liver metastases were subjected to immunofluorescent staining as indicated. Photographs were taken under fluorescence microscopy. I) human metastatic tumor burden in the livers of the mice was assessed with human-specific quantitative PCR (qPCR) as described in the section of “Materials and Methods” (n=6).

To investigate the effects of HMGB1 on A549 migration, invasion and metastasis both *in vivo* and *in vitro*, we used plasmid carrying shRNA to silence HMGB1 expression in A549 cell line. In the control group, A549 cells were transfected with plasmid carrying shRNA with no sequence homology to any known human genes. HMGB1 expression was effectively knocked down in the shRNA HMGB1 stable transfectant in comparison to that observed in shControl transfectant (Fig. 1A). The knockdown of HMGB1 in A549 cells led to an increase in spontaneous wound healing, while co-incubation of cells with rHMGB1 could inhibit the spontaneous wound healing. This result suggested that HMGB1 inhibited cancer cell migration and that this inhibition might be mediated by its released fraction (Figs. 1B and 1C). Consistently, the effect of HMGB1 in the regulation of cancer cell migration, as well as invasion, was extended in transwell cell migration assay (Fig. 1D). Moreover, HMGB1 silencing substantially increased A549 cell invasive ability in a transwell cell invasion assay, which is similar in phenomena seen in migratory cells (Figs. 1D and 1E). Again, rHMGB1 treatment primarily restored cell invasion inhibition in A549 shHMGB1 cells (Figs. 1D and 1E). To confirm that this effect is on migration/invasion rather than on cell growth, we also checked the cell proliferation rate in both cell lines. And no significant change on proliferation was observed due to the knockdown of HMGB1 (Fig. 1F). We next tested the role of HMGB1 expression in regulation of cancer cell metastasis in an *in vivo* animal model by observing the liver metastatic ability of A549 transfectants of shControl and A549 shHMGB1 by tail veins-injection of the transfectants into the 4-week-old nude mice. As shown in Fig. 1F, all mice with injections of either A549 shControl or A549 shHMGB1 cells displayed visible signs of metastatic liver tumor formation at 4 weeks post injection, in varying degrees (Fig. 1G, n=6). The metastatic tumors from both groups of mice were verified by IHC staining (Fig. 1H). To quantitative analyses of liver metastatic tumors, the quantitative real-time PCR was employed to measure the amount of human metastatic tumor cells within mouse lung tissues as described [16]. Briefly, this method targets for amplification a sequence of human genomic DNA found on the short arm of chromosome 12 (12p) that is not homologous to any region in the mouse genome. This target sequence does not lie within or in close proximity to any known gene. Human-specific primers amplify a 107-bp product from this locus but not from mouse tissue DNA, thereby specifically detecting the presence of human cancer cells residing in mouse liver tissues [16]. Results showed that human genomic DNA in livers from the mice injected with A549 shHMGB1 transfectant was more than 60-fold high than that observed from the mice injected with A549 shControl transfectant (*P* <0.01, n=6) (Fig. 1I). Thus, our results conclusively demonstrated that inoculated A549 cells were able to form metastatic tumors in nude mouse livers and that HMGB1 exhibited a marked inhibitory effect on the metastatic ability of A549 cells.

### Knockdown of HMGB1 specifically increased the β-Actin protein expression and Polymerization, and cytoskeleton formation

**Figure 2:**
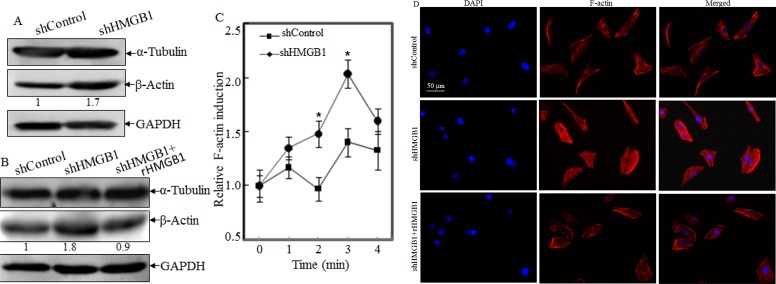
HMGB1 Knockdown increased the β-Actin protein expression and Polymerization, and cytoskeleon formation A) Protein levels of α-tubulin and β-actin in A549 (shControl) and A549 (shHMGB1) cells were determined by Western Blotting. GAPDH was used as protein loading controls. B) The cell extracts obtained from A549(shControl) and A549(shHMGB1) and A549(shHMGB1+rHMGB1) were subjected to Western Blotting as indicated. C) A549(shControl) and A549(shHMGB1) cells were exposed to EGF for indicated time and relative F-actin induction was determined and presented as described in “Materials and Methods”. D) A549(shControl), A549(shHMGB1) and A549(shHMGB1+rHMGB1) cells were stained with Oregon Green 488-conjugated phalloidin, and cytoskeleton and F-actin fibers were observed by confocal microscopy.

In mammalian cells, cytoskeleton is mainly composed of three distinct filaments: microfilament, microtubules, and intermediate filaments[17]. Among the three types, microfilament provides the primary mechanism for cell motility and is essential for most types of cell migration [5]. We therefore compared protein levels of β-actin and α-tubulin in A549 shControl and A549 shHMGB1cells. Results showed that β-actin expression levels were increased in HMGB1-shRNA cells in comparison to those in A549 shControl cells, while α-tubulin expression was comparable between the two transfectants under same experimental conditions (Fig. 2A). Again, rHMGB1 treatment could restore β-actin expression of A549 shHMGB1 cells to similar level in A549 shControl cells (Fig. 2B). In eukaryotic cells, actin exists as a globular monomer called G-actin or as a filamentous polymer called F-actin, a linear chain of G-actin subunits. Transformation between F-actin and G-actin is an important part of the cell mobility process [18]. Our most recently studies demonstrate that β-actin expression could be modulated at polymerization level by XIAP in HCT116 cells [19, 20]. To determine the potential involvement of HMGB1 in the regulation of actin polymerization, we treated both A549 shHMGB1 and A549 shControl cells with EGF, and then the dynamic alterations of F-Actin in both cells were determined. The results indicated that the dynamic induction of actin polymerization by EGF was observed in A549 shControl cells, whereas such alterations were significantly elevated in HMGB1-shRNA transfectant (Fig. 2C). Consistent with observed cell motility and actin polymerization, cell skeleton formation was elevated in A549 shHMGB1 cells in comparison to that in A549 shControl cells upon confocal microscopy investigation (Fig. 2D). Moreover, the increased filamentous actin was impaired by incubation of A549 shHMGB1 cells with rHMGB1 (Fig. 2D). These results suggested that HMGB1 downregulation of β-actin expression attributed to its abrogation of F-actin polymerization and cell skeleton formation, and thus inhibit cancer cell migration and invasion.

### Abrogation of HMGB1 elevated nWASP expression

**Figure 3:**
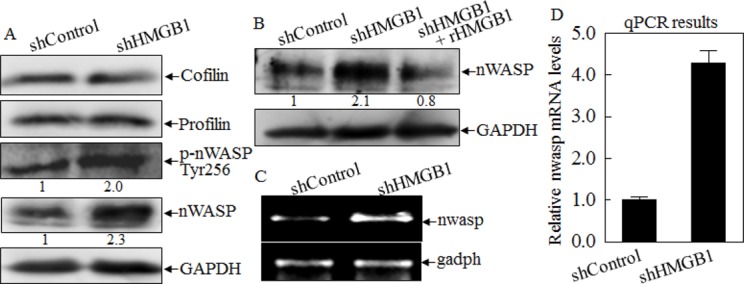
HMGB1 inhibited nWASP expression at both protein and mRNA levels A) nWASP protein expression and phosphorylation at Tyr256 in A549 (shControl) and A549 (shHMGB1) cells were evaluated by Western Blotting. GAPDH was used as a protein loading control. B) The cell extracts from A549(shControl) and A549(shHMGB1), A549 (shHMGB1+rHMGB1) were subjected to Western Blotting as indicated. C) RT-PCR was performed to determine the mRNA expression levels of *nWASP* in A549(shControl) and A549 (shHMGB1) cells. D) Real time PCR was performed using above samples to determine the quantitative change of nwasp mRNA expression.

As dynamic polymerization and depolymerization of actin filaments are vital processes for cell motility, they are precisely regulated by external and internal signaling events [5]. It has been reporter that actin polymerization is regulated by multiple proteins, including Cofilin, Profilin and nWASP [21, 22]. To elucidate the mechanisms underlying HMGB1 regulation of actin polymerization, we compared the expression and/or phosphorylation of cofilin, profilin, and nWASP between A549 shControl and A549 shHMGB1 transfectants. As shown in Fig. 3A, knockdown of HMGB1 expression markedly resulted in an increase of nWASP expression and a corresponding increase of phosphorylated nWASP at Tyr256, while it did not show any observable effects on expression of Cofilin and Profilin. The elevation of nWASP in A549 shHMGB1 cells could be reversed by incubation of cells with exogenous rHMGB1 in cell culture (Fig. 3B). The results obtained from RT-PCT indicated that knockdown of HMGB1 in A549 cells also led to 4 times increase in nwasp mRNA expression as compared to that in A549 shControl transfectant (Figs. 3C & 3D), suggesting that HMGB1could downregulate nWASP expression either at transcriptional level or mRNA stability.

### CREB phosphorylation and activation was inhibited by HMGB1 and was required for nWASP expression

**Figure 4:**
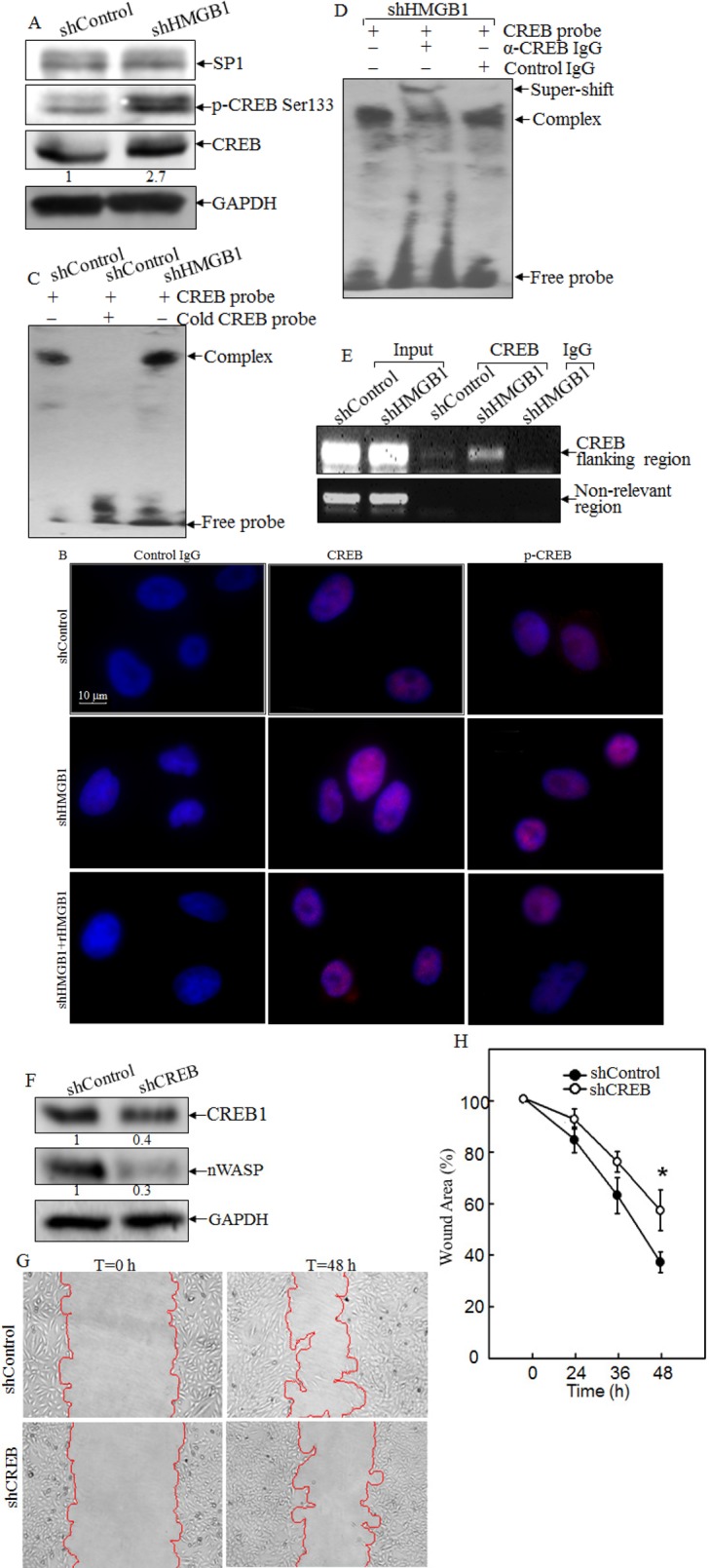
HMGB1 downregulated nWASP expression by inhibiting CREB phosphorylation and activation A) CREB protein expression and phosphorylation at Ser133 in A549(shControl) and A549(shHMGB1) cells were determined by Western Blotting. GAPDH was used as a loading control. B) A549(shControl), A549(shHMGB1) and A549 (shHMGB1+rHMGB1) cells were subjected to immunofluorescent staining using anti-CREB, or anti-phospho-CREB Ser133 antibodies. Photographs were taken under fluorescence microscopy. C & D) Nuclear extracts from A549(shControl) and A549(shHMGB1) cells were subjected to gel shift assay (C) and super gel shift assay (D) as indicated. E) Soluble chromatins of A549(shControl) and A549(shHMGB1) were prepared and subjected to ChIP assay using anti-CREB antibody as described in section of “ Materials and Methods”. F) Protein levels of CREB and nWASP, in A549(shControl) and A549(shCREB) cells were determined by using Western Blotting. GAPDH was used as a loading control. G & H) Cell migration behavior of A549(shControl) and A549(shCREB), were evaluated by wound-healing assay. Photographs were taken under microscopy (G), and the wound area was quantified using cell migration analysis software (H).

**Figure 5:**
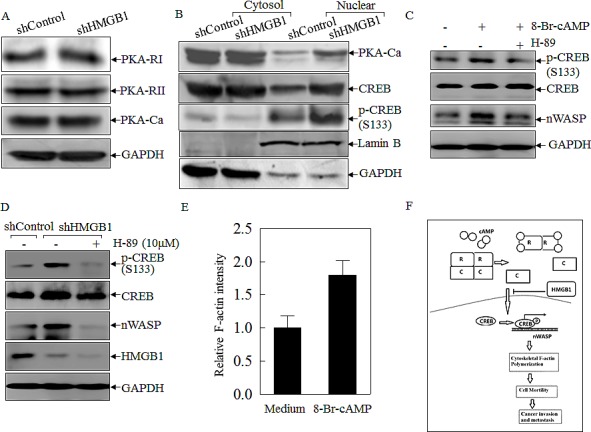
HMGB1 inhibited PKA-Ca and CREB nuclear localization in A549 cells A) Expression level of different subunits of PKA in A549(shControl) and A549(shHMGB1) cells were determined by Western Blotting. GAPDH was used as a loading control. B) A549 (shControl) and A549(shHMGB1) cells were extracted for isolation of protein fractionation using nuclear/cytosol fractionation kit. The isolated protein extracts were subjected to Western Blotting as indicated. Lamin B and GADPH were used as nuclear protein marker and cytoplasmic protein marker. C) A549 cells were pretreated with 10μM H-89 or control medium for 30 minutes, and then treated with 0.5mM 8-Br-cAMP or control medium for 6 hours. Expression level of CREB and nWASP were determined by Western Blotting. D) A549 (shControl) cells was cultured in control medium while A549 (shHMGB1) cells were treated with 10μM H-89 or control medium for 24 hours. Expression level of CREB, nWASP as well as CREB phosphorylation were determined by Western Blotting. E) A549 cells were pre-treated with 0.5mM 8-Br-cAMP or medium control for 6 hours, then were stimulated with EGF for 3min. Relative F-actin induction was determined. F) Anticipated molecular mechanisms for HMGB1 inhibition on cancer cell migration, invasion and metastasis.

To test the hypothesis that nWASP is regulated at the transcription level, putative transcription factors were predicted using the TFSEARCH software (http://www.cbrc.jp/research/db/TFSEARCH.html). The results showed that there were two potential transcription factors binding sites identified in the promoter region of *nwasp* gene, including SP-1 and CREB (Data not shown). SP-1 is a ubiquitously expressed transcription factor belonging to the family of C2H2-type zinc finger containing DNA-binding proteins [23]; while CREB is a β-ZIP transcription factor that activates target genes through cAMP response elements [24]. As shown in Fig. 4A, knockdown of HMGB1 profoundly increased the expression of CREB phosphorylation at Ser133 with a slight elevation of CREB protein expression in comparison to that in A549 shControl cells. We compared CREB nuclear localization and expression between A549 shHMGB1 and its parental vector control transfectant and observed that the increased CREB nuclear localization and expression in A549 shHMGB1 cells was reversed by incubation of the cells with exogenous rHMGB1, extending the inhibitory effect of HMGB1 on CREB expression and activation (Fig. 4B). To further observe the binding of CREB to *nwasp* DNA sequence, we carried out an EMSA assay to compare the CREB DNA binding activity between the two transfectants and found that HMGB1 silencing led to a marked increase in CREB binding activity, which was further verified by the results obtained from cold CREB probe competition assay and super-gel shift assay (Figs. 4C and 4D). Moreover, this notion was consistently supported by the data of a ChIP assay (Fig. 4E), showing that HMGB1 silencing markedly enhanced the recruitment of CREB to its binding site in n-wasp promoters, whereas control IgG and the primers targeting DNA sequence located at 1 kb upstream of the CREB binding site in the n-wasp promoter did not show observable products. Collectively, these results demonstrated that CREB phosphorylation and activation, as well as its binding activity to *nwasp* promoter region were specifically regulated by HMGB1 and might play a key role in nWASP upregulation in HMGB1 knockdown A549 cells. To provide direct evidence showing a critical role of CREB in nWASP expression and cancer cell migration, shRNA specifically targeting CREB was transfected into A549 cells. The stable transfectant, A549 shCREB and its vector control, A549 shControl, were established and identified, as shown in Fig. 4F. The knockdown of CREB not only lead to a dramatic reduction of nWASP expression in A549 cells, it also impaired A549 cell migration (Figs. 4G & 4H). These results strongly suggested that CREB was an HMGB1 downstream transcription factor responsible for regulation of n-WASP expression and cell migration. To identify the upstream kinase of CREB, we further tested the activation of PKA. As shown in Figures 5A & 5B, the expression of either the regulatory or the catalytic subunit of PKA in cell extracts does not show an observable change after HMGB1 knockdown. However, HMGB1 silencing markedly enhanced the nuclear translocation of both PKA-Cα and CREB, as well as the CREB phosphorylation on Ser133, demonstrating that PKA-Cα might be an upstream kinase mediating CREB activation. The inhibition on nWASP expression can be recovered by applying cAMP analog, 8-Br-cAMP, to the cell (Fig. 5C). Moreover, inhibiting the PKA activity by applying its inhibitor H-89 lead to less CREB phosphorylation as well as nWASP expression (Figs. 5C & 5D). The F-actin content assay also shows that that the dynamic induction of actin polymerization by EGF was elevated by 8-Br-cAMP treatment (Fig. 5E). By summarizing the findings above, we depicted the known mechanism of cAMP pathway as well as the involvement of HMGB1 in Figure 5F.

## DISCUSSIONS AND CONCLUSIONS

Due to the motivating forces as well as the structural bases which cancer metastases rely upon, the mechanistic cause of cell motility is a key step in the process of understanding cancer invasion and metastasis. Because they are potential drug targets of treatment to cancer metastasis, the molecules that participate in the regulation of cell motility have drawn significant attention in the cancer research field. In the current study, we examined the mechanisms underlying HMGB1 regulation of cancer cell motility from the upstream upstream kinases to final actin polymerization. We found that HMGB1 is a key regulator of CREB activation *via* inhibiting PKA-Cα nuclear localization. This function further reduces nWASP expression, and further results in a retarding effect upon actin polymerization as well as cytoskeleton formation, which makes HMGB1 a contributor to cancer cell motility, invasion and metastasis.

HMGB1 can interact with proteins including p53, p73, retinoblastoma protein (RB), and NFκB when it presents in the nucleus [25]. Those proteins are well-known and have proven to be key regulators of cancer progression [26, 27]. The finding outlined in elucidating the role of HMGB1 in regulation of cell motility is controversial [28-30]. In some study, the interaction between HMGB1 and its most important receptor, RAGE, was shown to facilitate the cancer cell migration [28]. However, higher expression of HMGB1 and RAGE does not always have poor prognosis of tumor development [29]. RAGE downregulation has also been reported to support non-small cell lung carcinoma [30]. In current studies, the findings that exogenous human recombinant HMGB1 successfully recovered the deficiency of HMGB1 production caused by knockdown of endogenous HMGB1 clearly show that a low level of HMGB1 in the cell environment caused by HMGB1 gene knockdown can be compensated for by adopting exogenous human recombinant HMGB1, indicating a regulatory role that HMGB1 plays in cancer cell migration, invasion and metastasis. It is known that nWASP binds to actin filaments and regulates cytoskeleton reorganization [31] and that HMGB1 is able to regulate Rho-GTPase activity [32]. Our current studies demonstrated that knockdown of endogenous HMGB1 increased A549 cancer cell migration is accompanied by induction of actin polymerization, cell skeleton formation and nWASP expression, suggesting that an inhibitory effect of HMGB1 on cancer cell migration and filament formation might be due to its regulation of nWASP expression. As actin acts as the basic structure and source of motile force of cell movement, our finding provides insight into the understanding of HMGB1 in the regulation of cancer cell motility, invasion, and metastasis. Moreover, we proved that the observed alteration on both cellular and protein level as a consequence of endogenous HMGB1 knockdown can be reversed by exposing the cell to human recombinant HMGB1. This indicates that the inhibitory effect on cell migration as well as its corresponding molecular mechanisms is dependent on the HMGB1 which exists in the microenvironment. Extracellular HMGB1 can be released from necrotic cells, apoptotic cells, autophagic cells, and tumor cells treated with chemotherapy, and those released HMGB1 can serve as ligands of receptors, including TLR-4 and RAGE [13-15, 33-35]. Although it is a nuclear protein, in conditions such as the activation of monocytes, HMGB1 can be secreted as a consequence of lysosome exocytosis [36]. Various studies have been made of developing the role of secreted HMGB1. Some of its functions were revealed as control of T cell activation and some inflammatory effect[37, 38]. Our finding discovered the effect of extracellular HMGB1 on actin polymerization, cell skeleton formation, and nWASP expression, as well as the consequent cell migration. Though question still remains about the exact mechanism of how environmental rHMGB1 triggers the series of alterations within the cell, we surmise it is very likely that HMGB1 serves as a signal molecule by interacting with its membrane receptors during this process. The receptors of HMGB1 includes RAGE and TLR-2/4/9. And the interactions between them lead to various consequences including MAPK phosphorylation, as well as activation of cdc42 and Rac protein [39]. However, the direct association between PKA-Cα and above signaling pathways has not been established yet, and will be our next goal of investigation.

Although previous studies on nWASP have mostly been focused on its intracellular function, its role in actin reorganization has also been recognized by some reports. Structural analysis shows that the VCA region of nWASP directly binds to monomeric actins and is essential to nucleate actin polymerization [40]. Deficiency of the *nwasp* gene has been associated with developmental delay in mouse embryos and even unviability, suggesting the importance of nWASP in cell growth [41]. The nWASP functional regulation is mostly achieved *via* its phosphorylation and conformational change by interaction with the Arp2/3 complex, however regulation of nWASP expression has not yet been explored or reported [42]. Our current studies showed that abrogation of HMGB1 expression by its specific shRNA resulted in upregulation of nWASP expression at both protein and mRNA levels, whereas exogenous rHMGB1 was able to inhibit nWASP expression, suggesting potential transcriptional modulation of the *nwasp* gene by HMGB1. This notion was further extended and supported by the findings obtained from identification of HMGB1 regulation of CREB cellular nuclear localization, binding to *nwasp* promoter region and CREB regulation of nWASP expression and cancer cell migration. CREB is the binding protein of DNA sequence cAMP response element (CRE). CREB mediates target gene transcription that is due to cAMP signal activation [43]. The results from gel shift assay, super shift assay and ChIP assay indicated that CREB was able to specifically bind to the promoter region of the *nwasp* gene, suggesting that CREB might be the transcription factor responsible for initiating *nwasp* mRNA transcription. This is consistently supported by results which showed that knockdown of CREB by its shRNA resulted in downregulation of nWASP expression as well as cell migration. When activated, cAMP binds to the regulatory subunits of PKA and releases its catalytic subunits. This allows the catalytic subunit to function within nucleus. The catalytic subunit PKA-Cα can phosphorylate CREB and increase its interaction with DNA as well as the transcription cofactors [44, 45]. Although this process has been known for decades, the regulation of PKA translocation is not fully understood. Some previous works indicated that failure of PKA catalytic subunit translocation largely impairs CREB phosphorylation [46]. Our results are consistent with the fact that translocation of the PKA-Cα subunit into the nucleus is required for CREB phosphorylation. Also, for the first time, we have shown participation of HMGB1 in this process. HMGB1 serves as a signaling molecule and interacts with various receptors, triggering different downstream pathways, which may have an effect on either the release of the PKA catalytic subunit, or the nucleus translocation of the released catalytic subunit. Further elucidation of molecular basis for HMGB1 regulation of PKA-Cα translocation is current undergoing project in our laboratory.

Overall, the results from current studies indicate that soluble HMGB1 inhibits nWASP expression by suppressing CREB activation *via* inhibition of PKA-Cα nuclear translocation, which in turn results in a retardant effect on actin polymerization and cytoskeleton formation, further contributing to inhibition of cancer cell motility, invasion and metastasis. These novel mechanisms, which underlie the functions of HMGB1 in regulation of cancer cell motility could provide information for utilization of soluble HMGB1 as a promising target for therapy of metastatic tumors or chemoprevention of metastatic tumor formation.

## MATERIALS AND METHODS

### Reagents

Recombinant human HMGB1 (rHMGB1) and H-89 was purchased from Sigma (St. Louis, MO). The small hairpin RNA (shRNA) specifically targeting human HMGB1 and their control plasmids were purchased from Open Biosystems (Pittsburgh, PA, USA). Lipofectamine 2000 was purchased from Invitrogen (Carlsbad, CA, USA). 8-Br-cAMP was from Santa Cruz Biotechnology (Santa Cruz, CA, USA). Transwell chamber was the product of Corning Company (Corning, NY, USA). Antibodies against CREB, CREB phosphorylation at Ser133, SP1, Cofilin, Profilin, nWASP, nWASP phosphorylation at Tyr256 or Ser484 were products from Cell Signaling Technologies (Beverly, MA). Antibodies against α-Tubulin, GFP, β-Actin, HMGB1, Lamin B, PKA RI, PKA RII, PKA-Cα, and GAPDH were purchased from Santa Cruz Biotechnology (Santa Cruz, CA, USA).

### Cell culture and transfection

A549 human adenocarcinoma cells (ATCC CCL-185) were cultured in DMEM containing 10% fetal bovine serum (FBS) supplemented with penicillin (50 units/ml), streptomycin (50 μg/ml), and sodium pyruvate (1 mM). Cell transfections were performed with Lipofectamine reagent (Invitrogen) or FuGENE® HD Transfection Reagent (Roche, Germany) according to the manufacturer's instructions. A549 was transfected with HMGB1-shRNA or its nonsense control shRNA, CREB-shRNA or its nonsense control shRNA, respectively, as described in our previous studies [47, 48]. For stable transfection, cultures were subjected to hygromycin B or G418 or puromycin drug selection, and survived cells from the antibiotic selection were pooled as stable mass transfectants. These stable transfectants were then cultured in the selected antibiotic-free medium for at least two passages before being used for experiments. The stable transfections obtained from the transfection mentioned above are named as A549 (shHMGB1), A549 (shCREB) and A549 (shVector), respectively.

### Wound healing assay

A549 cells suspended in 10% FBS DMEM medium were seeded into each well of 6-well plates and cultured until 80% confluence. Wounds were made by sterile pipette tips. Cells were washed with serum-free PBS and then cultured in 10% FBS DMEM medium for time points indicated in the Figure Legends. In this experiment, a group of A549 (shCREB) cells were treated with recombinant human rHMGB1. For this treatment, 10% FBS DMEM medium containing 1.0μg/mL rHMGB1 were used at the last step. Photographs reflecting the areas of wounds in the control group and test group were taken at specific time points, as indicated. The areas of wounds were quantified using cell migration analysis software (Muscale LLC., Scottsdale, AZ, USA).

### Cell migration-invasion assay

BD BioCoat^TM^ Matrigel^TM^ Invasion Chamber (BD Biosciences, MA, USA) was used for the invasion assay. Cells (2.5×10^4^) were seeded per insert in triplicates in 500μL serum-free DMEM medium with or without containing 1.0μg/mL rHMGB1. Inserts were placed in wells containing 500 μL medium with 5% FBS containing 20ng/mL 12-O-tetradecanoylphorbol-13-acetate (TPA). The cells were incubated for 36 and 72 hours in an incubator with 5% CO_2_ humidified atmosphere. The same invasion chamber, without matrigel, was used as the cell migration control. At the end of culture, cells on the upper surface of the filters were completely removed by wiping with a cotton swab. The membrane was cut with a sharp knife and cells on the lower surface were fixed, stained with crystal violet. The levels of invaded/migrated cells were determined using the CellTiter-Glo® Luminescent Cell Viability Assay (Promega, USA), according to manufacturer's instructions. Invasion (%) = (ATP activity of invaded cells /ATP activity of migrated cells) × 100%.

### Cell Proliferation Asssay

The cell proliferation were determined by using the CellTiter-Glo® Luminescent Cell Viability Assay (Promega, USA) according to manufacturer's instruction. Cell proliferation are expressed as percentage of time 0; Values are means ± SE in triplicate.

### *In Vivo* cancer metastasis assay

Tumor cells (10^6^ cells/mouse) were injected into the tail veins of each of six 4-week-old female nude mice (BALB/cABomCr-nu/nu; SLACCAS, Shanghai, P. R. China), which were cared for in accordance with the institutional guidelines. Six other mice were used as controls with both matched size and age. Four weeks after the injection, the mice were euthanized with diethyl ether, and the liver tissues were removed and examined microscopically for metastatic foci after fixation in 10% formalin solution. They were then processed for histological examination. Human metastatic tumor burden in the livers was assessed with human-specific quantitative PCR (qPCR) using DNA isolated from a homogenous mixture of both livers, according to the previously described approach [47], with qPCR primers: 12p F (5′-ggg aca gac act gag cct tga g-3′), 12p R (5′-tga ccc tga taa agt ttc ttg gaa-3′).

### Western blotting assay

Two×10^5^ cells were seeded into each well of 6-well plates and cultured until they reached 70-80% confluence. The cell extracts were subjected to Western blotting using specific antibodies, as indicated. The protein band, specifically bound to the primary antibody, was detected using an anti-rabbit IgG-AP-linked antibody and an ECF Western blotting system (Amersham Biosciences, Piscataway, NJ). Densitometry scan was made with Quantity One software by Bio-Rad.

### F-actin Content Assay

For quantification of F-actin in adherent A549 cells, cells were cultured in 10% FBS DMEM medium until they were 80-90% confluent. The medium was replaced with 0.1% FBS DMEM medium and incubated for 4 hours. Cells were then exposed to 25 ng/mL EGF for the time periods as indicated, and then fixed with 3.7% formaldehyde for 10 min in PBS and permeabilized with 0.1% Triton X-100 in PBS for another 10 min. After washing with PBS 3 times, the cells were blocked in 1% BSA/PBS at room temperature for 20 min, and then stained on a rotator with Oregon Green 488-phalloidin (1:40 in 1% BSA/PBS) for 30 min. Cells were washed with PBS again 3 times and the bound Oregon-phalloidin was extracted using 100% methanol at 4°C for 90 min. After extraction, the methanol was removed, plated cells were washed with PBS 3 times and a Bovine Carbonic Anhydrase assay was performed at 37°C to determine total cell protein in each sample. Fluorescence of the methanol extraction solution for each sample was recorded at 465 nm excitation and 535 nm emission, and normalized against total protein in each sample [49]. The results were expressed as relative F-actin content: F-actin(T_n_)/F-actin(T_0_)= [Fluorescence (T_n_)/ mg per mL] / [Fluorescence (T_0_)/ mg per mL].

### Isolation of Cellular Fraction

Cells (3-4×10^6^) were seeded into each of 10 cm dishes and cultured in 10% FBS DMEM medium for 2 days. We separated nuclear extract from cytoplasmic fraction of A549 cells by using Nuclear/Cytosol Extraction Kit (Biovision, CA, USA) using the previously described method [50]. Equal protein concentrations were determined using a protein quantification assay kit (Bio-Rad, CA, USA). Nuclear extracts were stored at 80 °C until they were used.

### Immunofluorescent Staining and Confocal Microscope

A549 cells and their transfectants were cultured on cover slides in 10% FBS DMEM medium for 48 hours. For EGF stimulation, the medium was replaced with 0.1% FBS DMEM medium and incubated for 4 hours, then treated with EGF (25ng/mL) for the times indicated. The cells were fixed with 3.7% paraformaldehyde for 15 min,e then permeabilized with 0.1% TritonX-100 in PBS for 15 min at room temperature. The cells were then blocked with 1% BSA/PBS for 30 min, and incubated with oregon-conjugated phalloidin for 30 min at room temperature, then stained with 0.1 μg/mL DAPI for 1 min. The slides were washed three times with PBS and mounted with antifade reagent (Molecular Probes). The cells were observed under a confocal microscope (Leica DMI6000B).

### Electrophoretic mobility shift assay (EMSA) and super gel shift

We performed the EMSA using the LightShift® Chemiluminescent EMSA Kit (Pierce, IL, USA) according to the manufacturer's instructions. Nuclear extracts were isolated with a Nuclear/Cytosol Fractionation Kit (BioVision, CA, USA). The specific probe pair designed for CREB was 5′-aca tga att tac gca aca aaa ata-3′ and 5′-tat ttt tgt tgc gta aat tca tgt-3′. The probe was conjugated with biotin by a Biotin 3′ End DNA Labeling Kit (Pierce, IL, USA) following the manufacturer's instructions. Nuclear protein (4 μg) was subjected to the gel shift assay by incubation with 1 μg of poly(dI-dC) DNA carrier in DNA binding buffer (10 mM Tris [pH 8.0], 150 mM KCl, 2 mM EDTA, 10 mM MgCl_2_, 10 mM dithiothreitol, 0.1% bovine serum albumin, 20% glycerol). The biotin-labeled double-stranded oligonucleotide (1μL) was then added, and the reaction mixture was incubated at room temperature for 50 min. For competition experiments, a 50-fold molar excess of the unlabeled double-stranded oligonucleotide was added before the addition of the labeled probe. For the super gel shift assay, nuclear extracts were incubated with 2 μg of antibody for 30 min at 4 °C before addition of the probe. DNA-protein complexes were resolved by electrophoresis on 5% nondenaturing glycerol-polyacrylamide gels. Luminescent signal was developed by a LightShift® Chemiluminescent EMSA Kit, and detected by an automatic developing machine (Kodak, MA).

### Chromatin immunoprecipitation (ChIP) assay

The ChIP assay was performed using the EZ ChIP kit (Upstate, NY, USA) according to the manufacturer's instructions. Briefly, cells (3~4×10^6^) were seeded into each of 10 cm dishes and cultured in 10% FBS DMEM medium for 2 days, and the cells were then treated with 1% formaldehyde for cross-linking the genomic DNA and the proteins. The cross-linked cells were suspended in lysis buffer and sonicated to generate 200- to 500-bp chromatin DNA fragments. After centrifugation, the supernatants were incubated with anti-CREB or the control normal rabbit IgG at 4 °C overnight. The immune-complex was captured by protein G agarose saturated with salmon sperm DNA and then eluted with the elution buffer. Cross-linked DNA-protein was heated at 65 °C for 4 hr, and the DNA was extracted and subjected to PCR analysis. To specifically amplify the region containing the putative CREB-responsive elements on the mouse *nwasp* promoter, we performed PCR using the following pair of primers: 5′-cag tct ggt tag tac atc ag-3′ (forward) and 5′- atc tca ctg ttc tcc aca -3′ (reverse). The primers targeting the region 1 kb upstream of the CREB binding site on the *nwasp* promoter were also used in the PCR analysis to support the specificity of the ChIP assay: 5′- tga tta ggg taa tta gca-3′ (forward) and 5′-gta gca ctg tag gat gac t-3′(reverse).

### Real-Time RT-PCR

Total RNA from each sample was isolated with RNeasy Mini Kit (Qiagen Inc., Valencia, CA). The reverse transcription reactions were conducted with Transcriptor First Strand cDNA Synthesis Kit (Roche, Indianapolis, IN). Real-time PCR with SYBR Green PCR Master Mix (Applied Biosystems, Foster City, CA) was performed using ABI Prism 7700 Sequence Detector (Applied Biosystems). The PCR primers were: 5′- tga tta ggg taa tta gca-3′ (forward) and 5′-gta gca ctg tag gat gac t-3′(reverse).

### Statistical analyses

Ordinary one-way ANOVA software was used to statistically determine the significance difference among each of experimental groups. If a significant difference was obtained by ANOVA analysis, the Tukey-Kramer multiple-comparisons t test was also used to verify the significance of the difference.
